# Supplementary material for: PhyloMed: a phylogeny-based test of mediation effect in microbiome
Source: Genome Biol. 2023 Apr 11;24:72. doi: 10.1186/s13059-023-02902-3 (PMC10088256; doi:10.1186/s13059-023-02902-3)
Supplement: Supplementary file 1 — Additional file 1. Note A: Definition of mediation effect via potential outcomes framework and identifiability assumptions; Note B: Details on obtaining $p_{\alpha}$pα and $p_{\beta}$pβ; Note C: Sobel’s test and joint significance test; Table S1-S6; Figure S1-S9. [file 13059_2023_2902_MOESM1_ESM.pdf]

PhyloMed: Supplementary Material

Note A: Definition of mediation effect via potential outcomes framework and identifiability assumptions

A.1: Regression models in Methods

In this section, we consider the regression models (1) and (2) in Methods. We drop the subject index i and node index j for the simplicity of notation and let G denote logit(M). The mediator regression model (1) can be rewritten as

$$E(G) = \boldsymbol{\alpha}_X^{\mathrm{T}}\mathbf{X} + \alpha T.$$

For the continuous and binary outcomes, regression model (2) can be rewritten as

$$E(Y) = \boldsymbol{\beta}_X^{\mathrm{T}}\mathbf{X} + \beta_T T + \beta G,$$

and

$$\text{logit}\{Pr(Y=1)\} = \boldsymbol{\beta}_X^{\mathrm{T}}\mathbf{X} + \beta_T T + \beta G.$$

Our primary interest is the natural indirect effect that measures the effect of the treatment on the outcome mediated through the mediator. The term has been defined and widely used in the causal inference literature and we use it interchangeably with mediation effect throughout this Supplementary Note. We first define the counterfactual notation. Let $G(T=t)$ be the potential outcome of the mediator that would have been observed if treatment T had been set to t. Let $Y_{T=t,G=g}$ be the potential outcome that would have been observed if treatment T and mediator G had been set to t and g, respectively. Note that $G(T=t)$ and $Y_{T=t,G=g}$ may or may not be observed. They are equivalent to observed values when their determinants are set to the observed values.

With these counterfactual notations, we can define the causal mediation effect (ME) as follows. For the continuous outcome, we have

$$\text{ME} = E\{Y_{T=t_1,G(T=t_1)}|\mathbf{X}\} - E\{Y_{T=t_1,G(T=t_0)}|\mathbf{X}\}.$$

For the binary outcome, the causal mediation effect is defined on the log odds ratio (OR) scale

$$\log\left(OR^{\text{ME}}\right) = \text{logit}\left[Pr\{Y_{T=t_1,G(T=t_1)} = 1|\mathbf{X}\}\right] - \text{logit}\left[Pr\{Y_{T=t_1,G(T=t_0)} = 1|\mathbf{X}\}\right].$$

These ME are expressed by conditioning on the measured confounders $\mathbf{X}$.

Identifying the mediation effect using the observed data requires five standard identifiability assumptions: (1) no unmeasured treatment-outcome confounders given $\mathbf{X}$; (2) no unmeasured mediator-outcome confounders given $(\mathbf{X}, T)$; (3) no unmeasured treatment-mediator confounders given $\mathbf{X}$; (4) no effect of treatment that confounds the mediator-outcome relationship; (5) no treatment and mediator interaction on the outcome.

Under these assumptions, ME for the continuous outcome can be expressed as

$$\begin{aligned}
&\text{ME} = E\{Y_{T=t_1,G(T=t_1)}|\mathbf{X}\} - E\{Y_{T=t_1,G(T=t_0)}|\mathbf{X}\}\\
=&\int E(Y|\mathbf{X}, T = t_1, G = g)f(G = g|\mathbf{X}, T = t_1)dg - \int E(Y|\mathbf{X}, T = t_1, G = g)f(G = g|\mathbf{X}, T = t_0)dg\\
=&\int (\boldsymbol{\beta}_X^{\text{T}}\mathbf{X} + \beta_T t_1 + \beta g)f(G = g|\mathbf{X}, T = t_1)dg - \int (\boldsymbol{\beta}_X^{\text{T}}\mathbf{X} + \beta_T t_1 + \beta g)f(G = g|\mathbf{X}, T = t_0)dg\\
=&\beta\left\{E(G|\mathbf{X}, T = t_1) - E(G|\mathbf{X}, T = t_0)\right\}\\
=&(t_1 - t_0)\alpha\beta.
\end{aligned}$$

For the rare binary outcome, we can approximate logit by log

$$\begin{aligned}
&\text{logit}\left[Pr\left\{Y_{T=t_1,G(T=t_0)} = 1|\mathbf{X}\right\}\right]\\
&\approx \log\left\{\int Pr(Y = 1|\mathbf{X}, T = t_1, G = g)f(G = g|\mathbf{X}, T = t_0)dg\right\}\\
&\approx \log\left\{\int \exp(\boldsymbol{\beta}_X^{\text{T}}\mathbf{X} + \beta_T t_1 + \beta g)f(G = g|\mathbf{X}, T = t_0)dg\right\}\\
&= \boldsymbol{\beta}_X^{\text{T}}\mathbf{X} + \beta_T t_1 + \beta(\boldsymbol{\alpha}_X^{\text{T}}\mathbf{X} + \alpha t_0) + \frac{1}{2}\beta^2\sigma^2,
\end{aligned}$$

where the last equation holds under the assumption that G follows the normal distribution with variance σ^2.

Then, the corresponding ME can be approximated as

$$\begin{aligned}\log\left(OR^{\mathrm{ME}}\right) &= \operatorname{logit}\left[Pr\left\{Y_{T=t_1,G(T=t_1)}=1|\mathbf{X}\right\}\right] - \operatorname{logit}\left[Pr\left\{Y_{T=t_1,G(T=t_0)}=1|\mathbf{X}\right\}\right]\\ &\approx (t_1-t_0)\alpha\beta.\end{aligned}$$

For the nonrare binary outcome, the ME can be approximated as

$$\begin{aligned}\log\left(OR^{\mathrm{ME}}\right) &= \operatorname{logit}\left[Pr\left\{Y_{T=t_1,G(T=t_1)}=1|\mathbf{X}\right\}\right] - \operatorname{logit}\left[Pr\left\{Y_{T=t_1,G(T=t_0)}=1|\mathbf{X}\right\}\right]\\ &\approx c(t_1-t_0)\alpha\beta,\end{aligned}$$

where $c = (1 + 0.35 \times \beta^2\sigma^2)^{-1/2}$.

In summary, for the continuous outcome, the null hypothesis of testing no causal mediation effect in the jth local model can be formulated as $H_0^j : \alpha_j\beta_j = 0$. For the binary outcome, if we are willing to make the normality assumption on $\operatorname{logit}(M_j)$, we can test the same null hypothesis no matter whether the binary outcome is rare or not.

A.2: An extension of the outcome model for the treatment-mediator interaction

In the presence of treatment-mediator interaction, we build the following outcome model

$$g\left\{E(Y)\right\} = \beta_X^{\mathrm{T}}\mathbf{X} + \beta_T T + \beta_1 G + \beta_2 TG.$$

Following the similar derivation in A.1, we can show that the mediation effect for the continuous outcome takes the form

$$\mathrm{ME} = (t_1-t_0)\alpha(\beta_1 + t_1\beta_2),$$

and the mediation effect for the binary outcome becomes

$$\log\left(OR^{\mathrm{ME}}\right) \approx c(t_1-t_0)\alpha(\beta_1 + t_1\beta_2),$$

where c is 1 for the rare outcome and $\{1+0.35\times(\beta_1+t_1\beta_2)^2\sigma^2\}^{-1/2}$ for the nonrare outcome. Therefore, given $t_1 = 1$, the null hypothesis of testing no mediation effect in the jth local model can be formulated as $H_0^j : \alpha_j(\beta_{j1} + \beta_{j2}) = 0$.

Note B: Details on obtaining p_α and p_β

To facilitate the description, we keep the subject index i and drop the node index j in this section. In PhyloMed, we need to obtain the p-value from testing $\alpha = 0$ in the mediator model and the p-value from testing $\beta = 0$ in the outcome model.

B.1: Asymptotic test p_α

In the mediator model, we only assume that the mean of G_i takes the form

$$E(G_i) = \boldsymbol{\alpha}_X^{\mathrm{T}} \mathbf{X}_i + \alpha T_i.$$

The parameters $\boldsymbol{\alpha}_X$ and α can be estimated by solving the estimating equation

$$\sum_{i=1}^{n} U(\boldsymbol{\alpha}_X, \alpha) = \sum_{i=1}^{n} (G_i - \boldsymbol{\alpha}_X^{\mathrm{T}} \mathbf{X}_i - \alpha T_i) \begin{bmatrix} \mathbf{X}_i \\ T_i \end{bmatrix} = \mathbf{0}.$$

To test the null hypothesis $\alpha = 0$, we use the generalized score statistic

$$U = \sum_{i=1}^{n} (G_i - \widehat{\boldsymbol{\alpha}}_X^{\mathrm{T}} \mathbf{X}_i) T_i,$$

where $\widehat{\boldsymbol{\alpha}}_X$ is the restricted estimate of $\boldsymbol{\alpha}_X$ under the null hypothesis. The empirical covariance estimate of U takes the form

$$V = \widehat{\mathbf{D}}_{22} - [\mathbf{Z}^{\mathrm{T}}\mathbf{Z}]_{21} [\mathbf{Z}^{\mathrm{T}}\mathbf{Z}]_{11}^{-1} \widehat{\mathbf{D}}_{12} - \widehat{\mathbf{D}}_{21} [\mathbf{Z}^{\mathrm{T}}\mathbf{Z}]_{11}^{-1} [\mathbf{Z}^{\mathrm{T}}\mathbf{Z}]_{12} + [\mathbf{Z}^{\mathrm{T}}\mathbf{Z}]_{21} [\mathbf{Z}^{\mathrm{T}}\mathbf{Z}]_{11}^{-1} \widehat{\mathbf{D}}_{11} [\mathbf{Z}^{\mathrm{T}}\mathbf{Z}]_{11}^{-1} [\mathbf{Z}^{\mathrm{T}}\mathbf{Z}]_{12},$$

where $\mathbf{Z}_i = (\mathbf{X}_i^{\mathrm{T}}, T_i)^{\mathrm{T}}$, $\mathbf{Z} = (\mathbf{Z}_1, \ldots, \mathbf{Z}_n)^{\mathrm{T}}$, $\widehat{\mathbf{D}} = \frac{n}{n-p+1} \sum_{i=1}^{n} (G_i - \widehat{\boldsymbol{\alpha}}_X^{\mathrm{T}} \mathbf{X}_i)^2 \mathbf{Z}_i \mathbf{Z}_i^{\mathrm{T}}$, p is the total number of regression parameters, and the matrix with subscript is the submatrix with the partition corresponding to $\boldsymbol{\alpha}_X$ and α.

We construct the test statistic U^2/V and obtain the asymptotic p-value p_α using the reference chi-square distribution with 1 degree of freedom.

B.2: Asymptotic test p_β

In the outcome model, we relate Y_i to $\mathbf{Z}_i = (\mathbf{X}_i^{\mathrm{T}}, T_i)^{\mathrm{T}}$ and G_i through a generalized linear model by specifying the conditional density function as

$$\prod_{i=1}^{n} \exp\left\{ \frac{Y_i\left(\boldsymbol{\gamma}^{\mathrm{T}}\mathbf{Z}_i + \beta G_i\right) - b(\boldsymbol{\gamma}^{\mathrm{T}}\mathbf{Z}_i + \beta G_i)}{a(\phi)} + c(Y_i, \phi) \right\}, \tag{1}$$

where $\boldsymbol{\gamma} = (\boldsymbol{\beta}_X^{\mathrm{T}}, \beta_T)^{\mathrm{T}}$ and β are regression parameters, ϕ is a dispersion parameter, and a, b, and c are specific functions. We use the first and second derivatives of b, which are denoted by b' and b''. For the linear regression model with error variance σ^2, we have $a(\phi) = \sigma^2$, $b(x) = x^2/2$, $b'(x) = x$, and $b''(x) = 1$. For the logistic regression model, we have $a(\phi) = 1$, $b(x) = \log(1 + e^x)$, $b'(x) = e^x/(1 + e^x)$, and $b''(x) = e^x/(1 + e^x)^2$.

To test the null hypothesis $\beta = 0$, we use the score statistic

$$U = \frac{1}{a(\widehat{\phi})} \sum_{i=1}^{n} \left\{ Y_i - b'(\widehat{\boldsymbol{\gamma}}^{\mathrm{T}}\mathbf{Z}_i) \right\} G_i,$$

where $\widehat{\boldsymbol{\gamma}}$ and $\widehat{\phi}$ are the restricted maximum likelihood estimators of $\boldsymbol{\gamma}$ and ϕ under the null hypothesis. Under H_0, U is asymptotically normal with mean 0 and variance

$$V = \frac{1}{a(\widehat{\phi})} \left[\sum_{i=1}^{n} b''(\widehat{\boldsymbol{\gamma}}^{\mathrm{T}}\mathbf{Z}_i) G_i^2 - \left\{ \sum_{i=1}^{n} b''(\widehat{\boldsymbol{\gamma}}^{\mathrm{T}}\mathbf{Z}_i) G_i \mathbf{Z}_i^{\mathrm{T}} \right\} \times \left\{ \sum_{i=1}^{n} b''(\widehat{\boldsymbol{\gamma}}^{\mathrm{T}}\mathbf{Z}_i) \mathbf{Z}_i \mathbf{Z}_i^{\mathrm{T}} \right\}^{-1} \times \left\{ \sum_{i=1}^{n} b''(\widehat{\boldsymbol{\gamma}}^{\mathrm{T}}\mathbf{Z}_i) \mathbf{Z}_i G_i \right\} \right],$$

We construct the test statistic U^2/V and obtain the asymptotic p-value p_β using the reference chi-square distribution with 1 degree of freedom.

B.3: Permutation test p_α and p_β

To obtain the permutation p-value, we employ Smith's permutation strategy. The p-value is calculated as the fraction of permutation test statistics that are as or more extreme than the observed one. Obtaining an accurate estimate of a small p-value requires a large number of permutations. However, most p-values are relatively large and can be estimated accurately with a small number of permutations. Thus, we employ an adaptive procedure that uses

small numbers of permutations for large p-values and large numbers of permutations only for small p-values. In addition, to estimate extremely small p-values, we adopt the approach, in which a generalized Pareto distribution (GPD) is fit to the extreme values of permutation test statistics. The detailed algorithm is provided in Algorithm 1.

Algorithm 1: Adaptive permutation procedure

input : B_{max} maximum number of permutations
output: P_{perm} permutation p-value

Determine R_{sel} for the desired precision level of p-value, which is calculated using the number of tests and the target FDR level;
$S_{obs} \leftarrow$ observed test statistic;
$S_B \leftarrow NULL, b \leftarrow 0, N_{exc} \leftarrow 0$;
while $N_{exc} < R_{sel}$ *and* $b < B_{max}$ **do**
    permute the orthogonalized covariate of interest with respect to **X** (Smith's method);
    $S_{perm} \leftarrow$ permutation test statistic;
    if $S_{perm} \geq S_{obs}$ **then**
        $N_{exc} \leftarrow N_{exc} + 1$;
    $S_B \leftarrow \{S_B, S_{perm}\}$;
    $b \leftarrow b + 1$;
end
if $b < B_{max}$ **then**
    $P_{perm} = N_{exc}/b$;
else
    if $N_{exc} \leq 10$ **then**
        $N_{gpd} \leftarrow 250$;
        $F(\cdot) \leftarrow$ empirical CDF of N_{gpd} most extreme S_B;
        $p_{gof} \leftarrow$ goodness-of-fit test p-value assessing whether $F(\cdot)$ follows GPD;
        while $p_{gof} \leq 0.05$ *and* $N_{gpd} > 0$ **do**
            $N_{gpd} \leftarrow N_{gpd} - 10$;
            Update $F(\cdot)$ and p_{gof};
        end
        if $p_{gof} > 0.05$ **then**
            $P_{perm} \leftarrow$ tail approximation using $F(\cdot)$;
        else
            $P_{perm} = (N_{exc} + 1)/(B_{max} + 1)$;
        end
    else
        $P_{perm} = N_{exc}/B_{max}$;
    end
end

Note C: Sobel's test and joint significance test

We compare the performance of proposed mixture-distribution-based method to traditional Sobel's and joint significance tests. In this section, we describe how we employ these two tests to obtain the p-value for testing the subcomposition mediation effect in each local model.

C.1: Sobel's test

In each local mediation model at the jth internal node, the Sobel test statistic takes the form

$$T_{Sobel,j} = \frac{\widehat{\alpha}_j \widehat{\beta}_j}{\sqrt{\widehat{\beta}_j^{\,2} \widehat{\sigma}^2_{\alpha_j} + \widehat{\alpha}_j^2 \widehat{\sigma}^2_{\beta_j}}},$$

where $\widehat{\alpha}_j$ is the estimates of the α_j under the mediator model (Equation (1) in Methods) and $\widehat{\beta}_j$ is the estimates of the β_j under the outcome model (Equation (2) in Methods). The $\widehat{\sigma}^2_{\alpha_j}$ and $\widehat{\sigma}^2_{\beta_j}$ are the estimated variances of α_j and β_j, respectively. The standard normal distribution is used as the reference distribution to calculate the p-value of $T_{Sobel,j}$.

C.2: Joint significance test

The joint significance test statistic takes the maximum of the permutation test p-values p_{α_j} and p_{β_j} (Note B.3). The usual uniform distribution for a null p-value is used to declare significance.

Additional Tables and Figures

Table S1: Empirical FDR in identifying mediating nodes when different mediation tests are employed in local models (target FDR = 0.05). PhyloMed (permutation version): mixture-distribution-based test; JS: joint significance test; Sobel: Sobel's test.

n	Num. mediating taxa	**PhyloMed**	**JS**	**Sobel**
**Continuous outcome**				
50	3	0.017	0	0
50	6	0.008	0	0
200	3	0.017	0	0
200	6	0.009	0	0
**Binary outcome**				
50	3	0.015	0	0
50	6	0.007	0	0
200	3	0.009	0	0
200	6	0.007	0	0

Table S2: Discovery rate in identifying the most recent common ancestor of mediating OTUs when different mediation tests are employed in local models (target FDR = 0.05). PhyloMed (permutation version): mixture-distribution-based test; JS: joint significance test; Sobel: Sobel's test.

n	Num. mediating OTUs	**PhyloMed**	**JS**	**Sobel**
**Continuous outcome**				
50	3	0.295	0.141	0.085
50	6	0.248	0.111	0.068
200	3	0.581	0.456	0.419
200	6	0.560	0.443	0.402
**Binary outcome**				
50	3	0.101	0.016	0
50	6	0.077	0.014	0
200	3	0.334	0.175	0.105
200	6	0.315	0.170	0.101

Table S3: Empirical type I error of the PhyloMed asymptotic global mediation test when different subcomposition models were used. LR: log-ratio model (proposed); BB: Beta-binomial model; QL: quasi-likelihood model for composition counts. The results were generated under the simulation setting for the continuous outcome and large sample size $n = 200$. The $|\mathcal{S}_\alpha|$ and $|\mathcal{S}_\beta|$ denote the number of treatment-associated OTUs and outcome-associated OTUs, respectively. Different combinations of ($|\mathcal{S}_\alpha|$, $|\mathcal{S}_\beta|$) represent different mixtures of mediation nulls H_{00}, H_{10} and H_{01}.

$\|S_\alpha\|$	$\|S_\beta\|$	**LR**	**BB**	**QL**
0	0	0.023	0.032	0.012
3	0	0.030	0.038	0.020
6	0	0.035	0.042	0.024
0	3	0.032	0.066	0.024
0	6	0.040	0.089	0.025

Table S4: Type I error and power of PhyloMed global test at the significance level of 0.05 when different pseudocounts ("psc") were added in the data. The simulation is conducted under the continuous outcome. The $|\mathcal{S}_\alpha|$ and $|\mathcal{S}_\beta|$ denote the number of treatment-associated OTUs and outcome-associated OTUs, respectively. The power results are under $|\mathcal{S}_\alpha| = |\mathcal{S}_\beta| = 3$ or 6 and the rest are the type I error results.

			**PhyloMed.A**			**PhyloMed.P**		
n	$\lvert\mathcal{S}_\alpha\rvert$	$\lvert\mathcal{S}_\beta\rvert$	psc=0.1	psc=0.5	psc=1	psc=0.1	psc=0.5	psc=1
50	0	0	0.019	0.020	0.021	0.027	0.028	0.026
50	3	0	0.022	0.021	0.021	0.030	0.028	0.028
50	6	0	0.034	0.036	0.035	0.044	0.042	0.042
50	0	3	0.020	0.021	0.021	0.026	0.025	0.024
50	0	6	0.024	0.024	0.023	0.031	0.028	0.029
50	3	3	0.517	0.468	0.448	0.533	0.495	0.470
50	6	6	0.705	0.664	0.643	0.727	0.682	0.665
200	0	0	0.024	0.023	0.024	0.027	0.025	0.024
200	3	0	0.029	0.030	0.030	0.033	0.032	0.035
200	6	0	0.034	0.035	0.034	0.038	0.036	0.037
200	0	3	0.034	0.032	0.031	0.035	0.034	0.034
200	0	6	0.039	0.040	0.042	0.042	0.043	0.041
200	3	3	0.807	0.790	0.780	0.812	0.794	0.783
200	6	6	0.963	0.954	0.948	0.965	0.955	0.950

Table S5: The p-value of global mediation tests in real data analyses using different pseudocounts

**Study**	**Pseudocount**	**PhyloMed.A**	**PhyloMed.P**	**MedTest***	**MODIMA***
Mouse cecal	0.1	0.053	0.064	0.452	1.000
	0.5	0.094	0.085	0.239	1.000
	1	0.141	0.126	0.683	1.000
Human gut	0.1	0.071	0.049	0.509	0.342
	0.5	0.074	0.047	0.676	0.267
	1	0.091	0.057	0.763	0.412

* Pseudocount is used in the calculation of the Aitchison distance

Table S6: Bias and standard deviation of the estimated proportions of the three null hypotheses using two different approaches described in Methods. The estimates are based on the asymptotic p-values p_{α_j}'s and p_{β_j}'s. The results were generated under the simulation setting for the continuous outcome and sample size $n = 200$. The $|\mathcal{S}_\alpha|$ and $|\mathcal{S}_\beta|$ denote the number of treatment-associated OTUs and outcome-associated OTUs, respectively. Mediation signals are present at some internal nodes under $|\mathcal{S}_\alpha| = |\mathcal{S}_\beta| = 3$ or 6 and the remaining settings are under the global mediation null.

$\lvert\mathcal{S}_\alpha\rvert$	$\lvert\mathcal{S}_\beta\rvert$	π_{00}		π_{10}		π_{01}	
		product	maxp	product	maxp	product	maxp
0	0	-0.062 (0.062)	-0.063 (0.064)	0.032 (0.046)	0.033 (0.047)	0.028 (0.043)	0.029 (0.045)
3	0	0.019 (0.074)	0.017 (0.077)	-0.050 (0.059)	-0.048 (0.061)	0.029 (0.043)	0.031 (0.046)
6	0	0.068 (0.080)	0.065 (0.083)	-0.100 (0.069)	-0.097 (0.070)	0.029 (0.042)	0.032 (0.046)
0	3	-0.010 (0.102)	-0.013 (0.107)	0.030 (0.042)	0.033 (0.047)	-0.023 (0.091)	-0.020 (0.094)
0	6	-0.008 (0.120)	-0.014 (0.125)	0.028 (0.039)	0.033 (0.047)	-0.025 (0.114)	-0.019 (0.116)
3	3	-0.117 (0.079)	-0.120 (0.084)	0.055 (0.048)	0.058 (0.052)	0.059 (0.062)	0.062 (0.066)
6	6	-0.190 (0.089)	-0.198 (0.098)	0.076 (0.048)	0.084 (0.054)	0.104 (0.075)	0.112 (0.081)

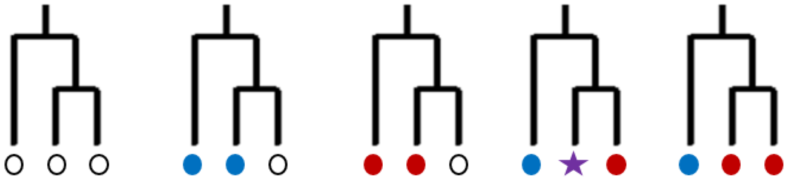

Presence of mediation effect on any of the three taxa at the leaves	*No*	*No*	*No*	*Yes*	*No*
Presence of mediation effect at the upper nodes after aggregating the leaf-level taxa	*No*	*No*	*No*	*Yes*	*Yes*

Fig. S1 Different scenarios of the ground truth of the presence/absence of mediation effects at the leaf-level taxa and the aggregated taxa at the common ancestors. White circles represent leaf-level taxa not associated with the treatment and outcome. Blue circles represent leaf-level taxa only associated with the treatment. Red circles represent leaf-level taxa only associated with the outcome. Purple star represents mediating leaf-level taxon associated with both the treatment and the outcome.

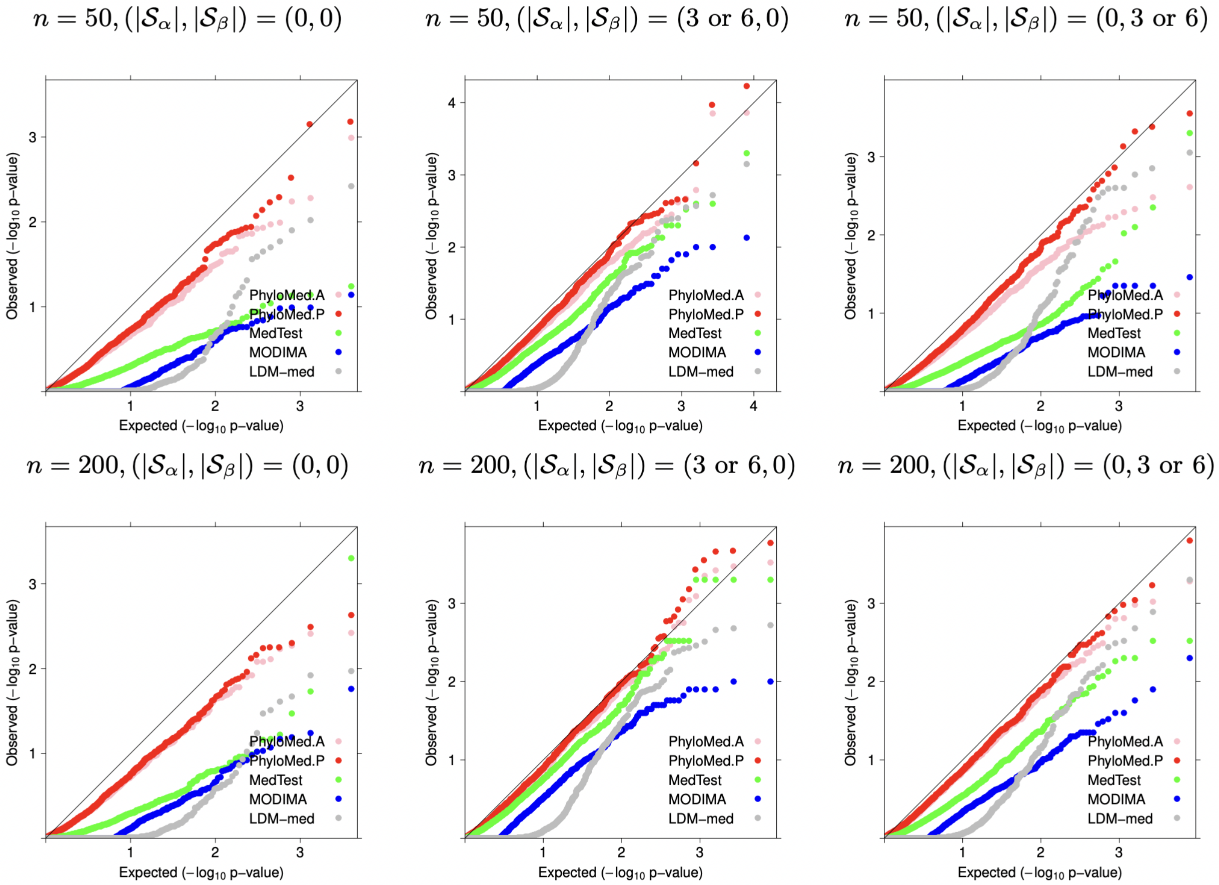

Fig. S2 Quantile-quantile plots of p-values from different global mediation tests in the simulation study for the binary outcome. The observed p-values were compared to the expected quantiles generated by the uniform null distribution. The $|\mathcal{S}_\alpha|$ and $|\mathcal{S}_\beta|$ denote the number of treatment-associated OTUs and outcome-associated OTUs, respectively. Different combinations of $(|\mathcal{S}_\alpha|, |\mathcal{S}_\beta|)$ represent different mixtures of mediation nulls H_{00}, H_{10} and H_{01}.

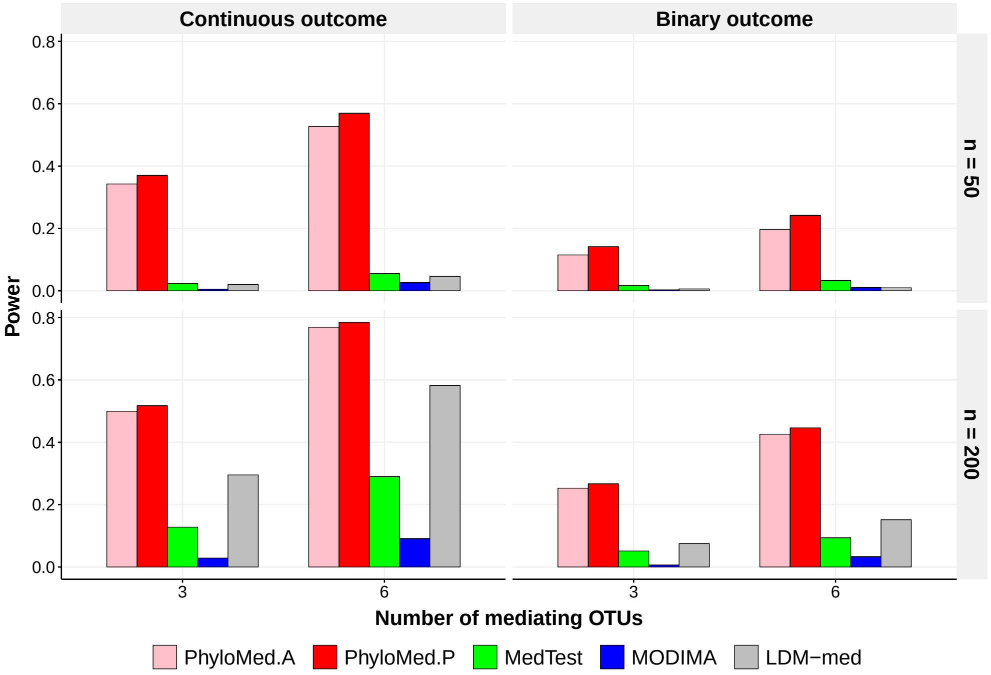

Fig. S3 Power comparison of different global mediation tests when including all OTUs in the basis dataset in the simulation. The mediating OTUs are clustered on the tree.

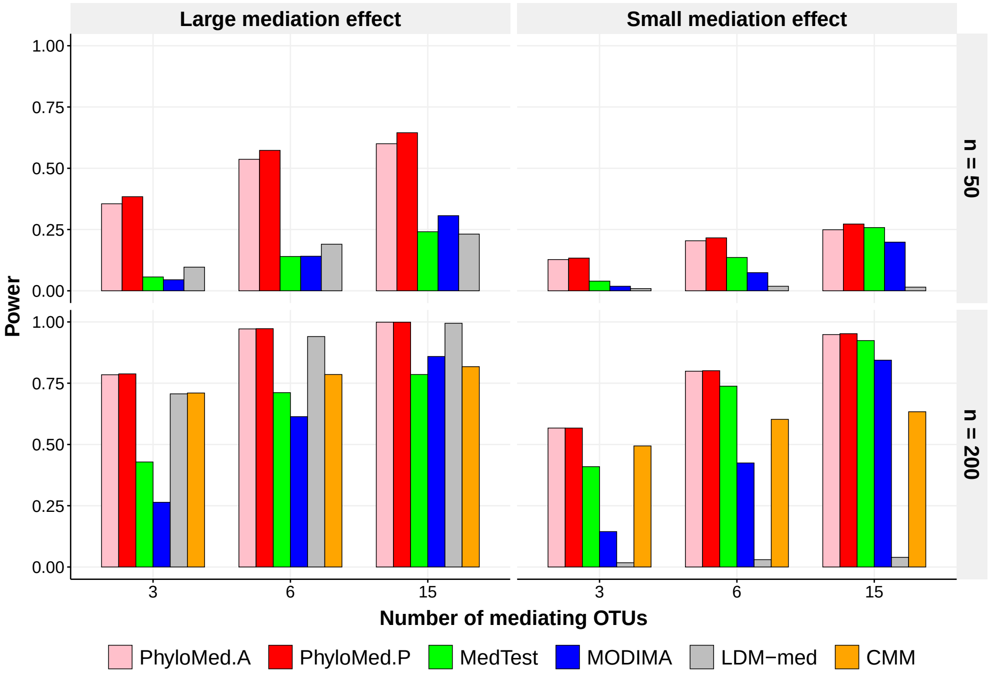

Fig. S4 Power comparison of different global mediation tests when mediating OTUs are randomly scattered. To further challenge the performance of PhyloMed, we added additional settings with 15 mediating OTUs and the small mediation effect. The outcome is continuous in this set of simulations.

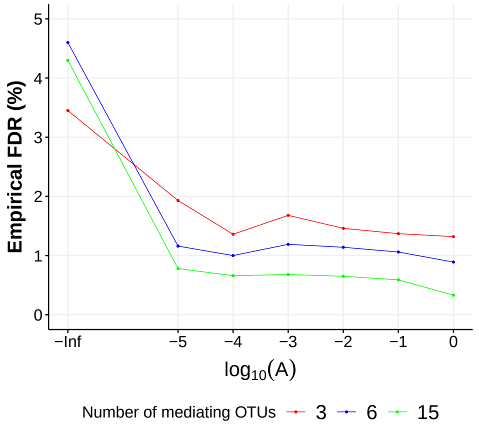

Fig. S5 Empirical FDR of PhyloMed (permutation version) with varying signal strength and density. The target FDR is 5%. The effect of mediator-outcome association is fixed and the effect of treatment-mediator association is controlled by A and increases from 0 to 1. The outcome is continuous and the sample size is 200 for this set of simulation.

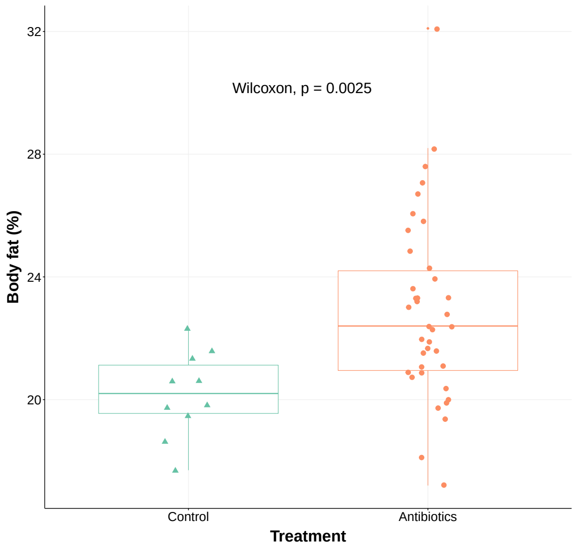

Fig. S6 Box plots of body fat percentage in the control and antibiotics treatment groups in the mouse cecal microbiome study.

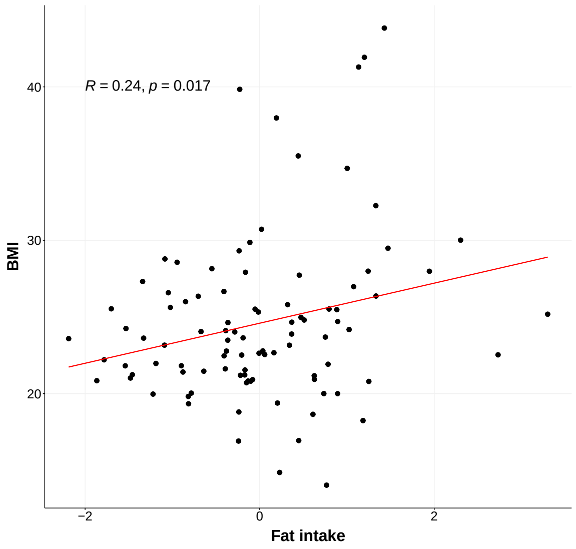

Fig. S7 Scatter plot of fat intake (after adjusting for total calorie intake) and BMI values in the human gut microbiome study.

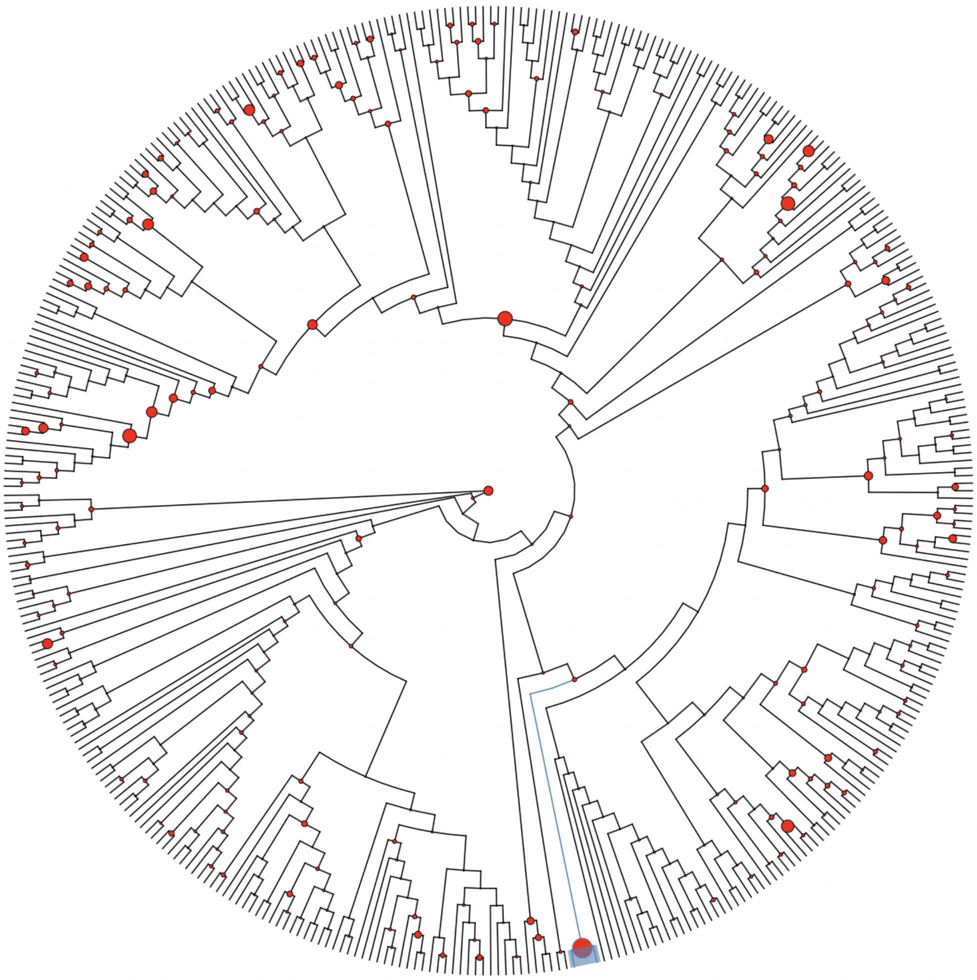

Fig. S8 Phylogenetic tree in the human gut microbiome data analysis. The size of the circle at each internal node is proportional to $-\log 10$(PhyloMed local mediation test p-value). The identified mediating internal node has the largest red circle and the subtree under the node with two OTU descendants is highlighted in a blue rectangle.

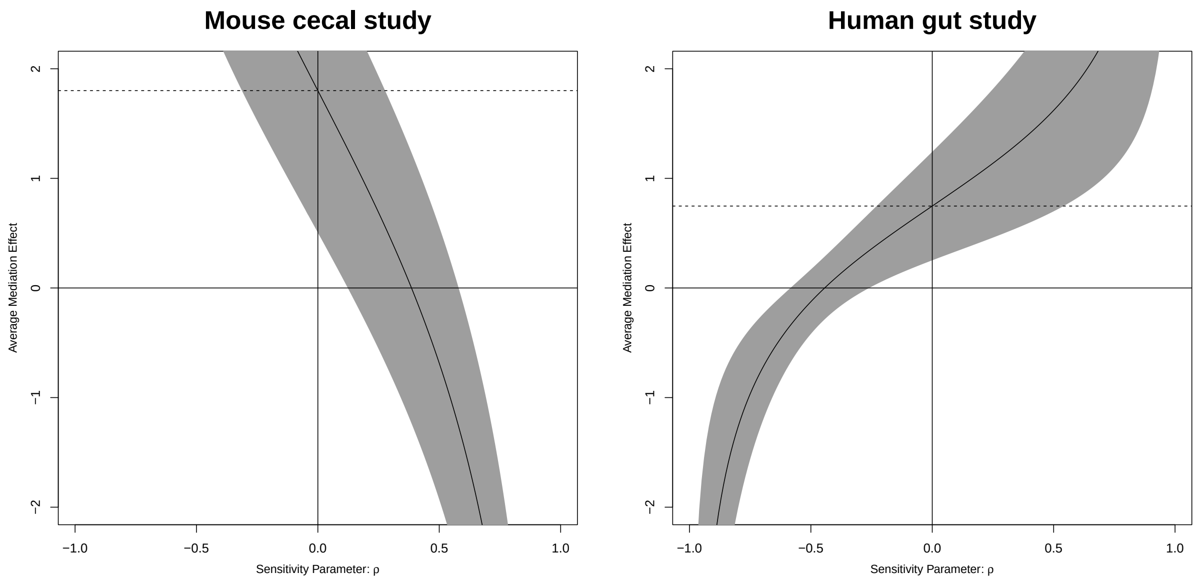

Fig. S9 Mediation sensitivity analysis for average mediation effect at the two identified mediating nodes in the mouse cecal microbiome study and human gut microbiome study, respectively. In each plot, the solid line represents the estimated average mediation effect for the identified subcomposition mediator (in log-ratio) for varying values of the sensitivity parameter ρ. The gray region represents the 90% confidence interval.
